# Risk factors for cognitive dysfunction and glycemic management in older adults with type 2 diabetes mellitus: a retrospective study

**DOI:** 10.1186/s12902-023-01476-2

**Published:** 2023-10-11

**Authors:** Fanyuan Ma, Qian Zhang, Juan Shi, Shuaifeng Li, Liping Wu, Hua Zhang

**Affiliations:** 1 Department of Geriatrics, Tangdu Hospital, Air Force Medical University, Xi’an, 710038 China; 2 Department of Geriatrics, Xijing Hospital, Air Force Medical University, Xi’an, 710032 China; 3 Department of Anatomy, Histology and Embryology, Air Force Medical University, Xi’an, 710032 China; 4 Department of Spine Surgery, General Hospital of PLA Tibet Military Area Command, Lhasa, 850007 China

**Keywords:** Cognitive dysfunction, Type 2 diabetes mellitus, Glycemic management, Dementia

## Abstract

**Background:**

Epidemiological evidence shows a robust relationship between cognitive dysfunction and type 2 diabetes mellitus (T2DM). This study identified major risk factors that might prevent or ameliorate T2DM-associated cognitive dysfunction in the realm of clinical practice.

**Methods:**

Using Mini-mental State Examination (MMSE) in the light of education level, we identified older adults with T2DM on admission aged 50 and above. We conducted this case–control study when eligible participants were divided into Cognitively Normal (CN) group and Cognitively Impaired (CI) group. Analytical data referred to demographic characteristics, clinical features, fluid biomarkers, and scale tests.

**Results:**

Of 596 records screened, 504 cases were included in the final analysis. Modified multivariate logistic regression analysis verified that homocysteine (OR = 2.048, 95%CI = 1.129–3.713), brain infarction (OR = 1.963, 95%CI = 1.197–3.218), dementia (OR = 9.430, 95%CI = 2.113–42.093), education level (OR = 0.605, 95%CI = 0.367–0.997), severity of dependence (OR = 1.996, 95%CI = 1.397–2.851), creatine kinase (OR = 0.514, 95%CI = 0.271–0.974) were significant risk factors of incident T2DM-related cognitive dysfunction in patients of advanced age.

**Conclusion:**

Our study supported a robust relationship between T2DM and cognitive dysfunction. Our results provide clinicians with major risk factors for T2DM-related cognitive dysfunction, in particular the protective role of creatine kinase.

## Introduction

Diabetes mellitus (DM), cognitive dysfunction and dementia may generally coexist in patients aged older than 65-year-old [1]. The presence of DM accelerating brain aging with cognitive deficits appreciably becomes apparent, and several lines of evidence suggest a more complex interaction between DM processes and cognitive dysfunction. Cognitive dysfunction affects many perspectives of routine life. It is believed that decline in cognitive function is associated with worse DM management, more recurring severe hypoglycaemic episodes, longer duration of DM, and perhaps more likelihood to suffer from cardiovascular events [2]. Yet, so far there does not appear to be a causal relationship between DM and cognitive decrements, despite the fact that DM is connected with cerebrovascular disease and an increased risk of stroke [3].

The coexistence of the condition possibly shares a common set of risk factors, principally hypertension and stroke but also alcoholic consumption, hyperlipemia, depression or anxiety, lower educational level, and physical inactivity. The individuals with DM suffering from brain dysfunction may be attributed to the poor management of glycemic control, identified as age at onset of DM, type of DM, duration of DM, and the presence of comorbidity [4]. DM is a chronic metabolic disease that is thought to affect the severity of neurocognitive dysfunction across an extended period of time, possibly through increased serum Aβl-42 levels, decreased adiponectin levels and inflammation reaction [5]. Meanwhile, advanced age and frailty pose unique challenges especially in the presence of DM-related cognitive dysfunction, such as falls, chronic pain, incontinence, and adverse effects of anti-diabetic medications [6].

Guideline for the prevention and treatment of type 2 diabetes mellitus (T2DM) in China (2020 edition) suggested a far more integrated management strategy that required monitoring several indexes to devise a personalized treatment regimen, where the goal of regimens is to obtain the most optimal glycemic control, and maintain glycated hemoglobin (HbA_1c_) values as close to the normal range as possible. It is suspected that higher HbA_1c_ levels at follow-up are associated with worse performance in cognition, despite whether DM developed [7]. Moreover, individuals experiencing longer duration of DM are likely to get into more episodes of recurrent severe hypoglycemia, and more drastic fluctuations when passing through several DM stages in blood glucose across the lifespan [8], contributing to a gradual decline in cognition. What's more, important sources of bias that should be taken into account when interpreting results of the research include a low level of education; depression or anxiety; and so-called cognitive mimics like tumors or rare neurological diseases.

The thyroid dysfunction was considered a potentially reversible cause of cognitive decline, and thyroid function was described as an essential component of the workup [9]. A previous study claimed that serum levels of liver biomarkers were correlated with psychiatric disorders or cognitive deficits, and Amyloid PET, CSF amyloid, Plasma amyloid, CSF phosphorylated tau [10], Apolipoprotein-B might influence the severity of cognitive deficits [11]. Additional features shared with the dystroglycanopathies include raised CK levels and variable mild cognitive delay [12]. Considering hemostasis and thrombosis might be important contributors to cognitive decline and dementia [13], evidence for the association of circulating hemostatic variables and dementia or cognitive impairment was collected.

## Methods

### Population

**Fig. 1:**
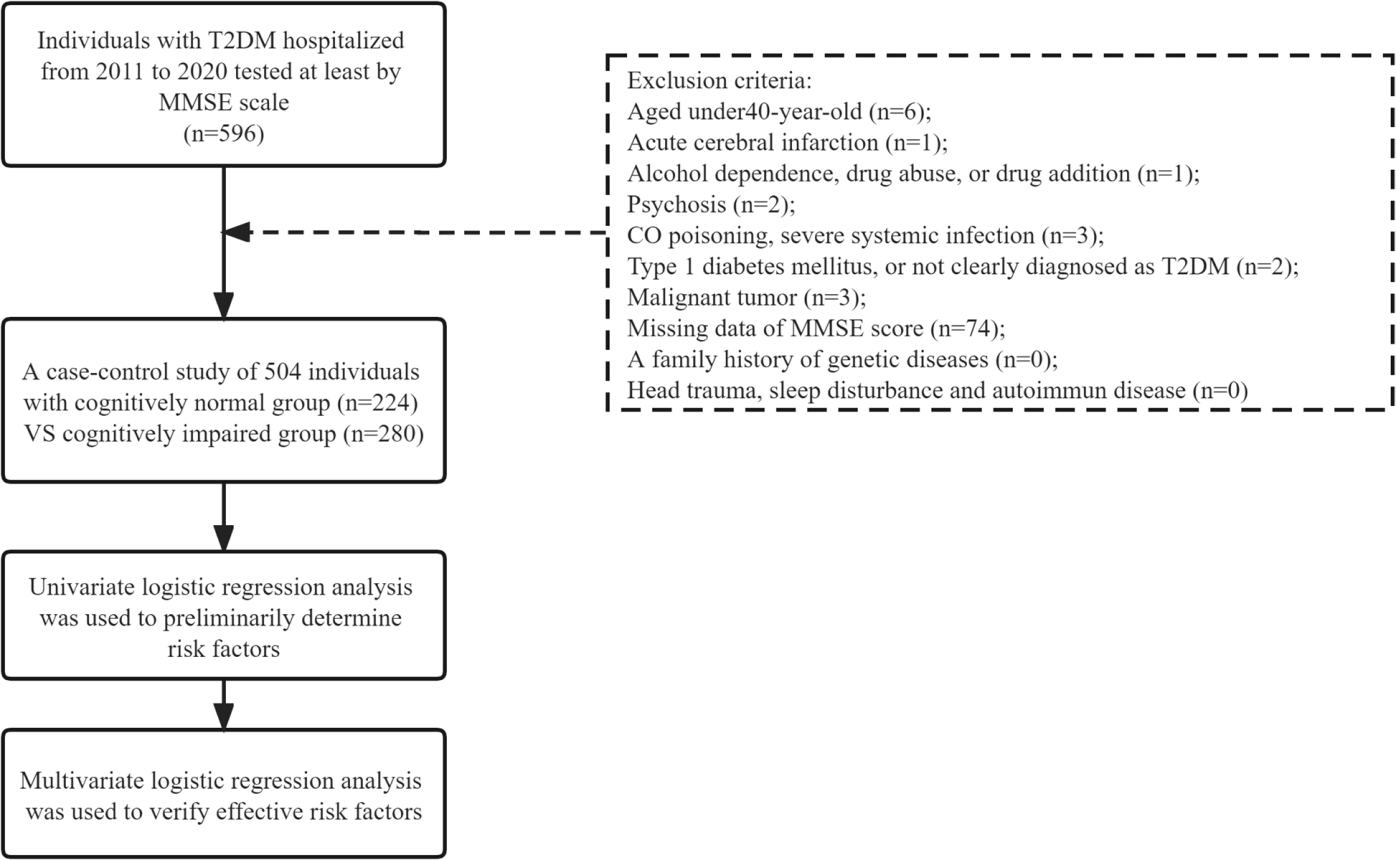
Flow chart of the study included and excluded

From the database in Xijing hospital, we gathered clinical and laboratory information of inpatients diagnosed with T2DM who were hospitalized from January, 2011 to December, 2020, and eventually we identified 504 enrolled individuals having been tested by Mini-mental State Examination (MMSE) scale (Shown in Fig. 1). The participants in the two groups were aged over 40, regardless of female and male (considering the gender radio balance), and the educational level ranged from illiteracy, primary school to junior high school or above.

The inclusion criteria for Cognitively Normal group (as CN group) were as follows: 1) diagnosed as T2DM or having a history of T2DM; 2) tested at least by MMSE; 3) normal range of MMSE score. The inclusion criteria of Cognitively Impaired group (as CI group) were as follows: 1) diagnosed as T2DM or having a history of T2DM; 2) tested at least by MMSE; 3) abnormal range of MMSE score.

The exclusion criteria of two groups were as follows: 1) aged under 40; 2) acute cerebral infarction; 3) alcohol dependence or drug abuse, drug addiction; 4) psychosis; 5) CO poisoning, severe systemic infection; 6) type 1 diabetes mellitus, or not clearly diagnosed as T2DM; 7) malignant tumor; 8) missing data of MMSE; 9) a family history of genetic diseases; 9) head trauma, sleep disturbance and autoimmun disease.

### Testing scale

The Chinese version of MMSE is a clinical questionnaire whose full mark shall be 30 points, and it consists of 7 items to assess time orientation, location orientation, immediate memory, attention and computation, delayed memory, language ability, visual space [14]. The standardized evaluations to verify cognitive impairment, which are amended for educational level according to scales of our hospital, are as follows: A MMSE scored < 17 for illiteracy; a MMSE scored < 20 for primary school level; a MMSE scored < 24 for junior high school and above. It is acknowledged that Montreal Cognitive Assessment (MoCA) is a reliable screening tool for retesting with superior sensitivity to the presence of cognitive impairment [15]. However, as most of the participants targeted in this study are are poorly educated, and some are even illiterate, MMSE based on educational background is more appropriate. In effect, MoCA seemed to be infrequently used with a large portion of censored data (65.67%) in terms of the sample [16]. The dependence status is ranged from self-care (91–100 points), mild dependence (61–90 points), moderate dependence (21–60 points) to severe dependence (0–20 points) on the base of Barthel Index [17]. The BMI number and stratification for Asian population are listed as: Severely underweight—BMI < 16.5 kg/m^2^; Underweight—BMI < 18.5 kg/m^2^; Normal weight—BMI < 23 kg/m^2^; Overweight—BMI < 25 kg/m^2^; Obesity—BMI greater than 25 kg/m^2^ [18].

### Clinical and laboratory data

We collected data for demographic and clinical complication characteristics, including gender, residence, admission age, education level, alcohol consumption, smoking, and the presence of hypertension, coronary heart disease, brain infarction, encephalatrophy, dementia, anxiety disorder, depression. In like manner, clinical scales like Hamilton Anxiety Scale (HAMA), Hamilton Depression Scale (HAMD), and severity of dependence in accordance with Barthel Index (BI) were measured. As for laboratory data, we gathered information on inflammation, liver and renal function, thyroid function, coagulation markers, and additional homocysteine (HCY), vitamin B_12_, folic acid, neuron specific enolase (NSE), which were thought to be related to the occurrence of cognitive dysfunction. Notably, integrated control objectives of T2DM in China (Shown in Table 1) are also involved.

**Table 1:** Integrated control objectives ofT2DM in China (2020 Edition)

Characteristics	Target value
Fasting Blood Glucose	4.4–7.0
Postprandial Blood Glucose	< 10.0
HbA_1c_ (%)	< 7.0
Blood Pressure (mmHg)	< 130/80
Total Cholesterol (mmol/L)	< 4.5
Triglyceride (mmol/L)	< 1.7
High-Density Lipoprotein Cholesterol (mmol/L)	
Male	> 1.0
Female	> 1.3
Low-Density Lipoprotein Cholesterol (mmol/L)	
Uncombined ASCVD	< 2.6
Combined ASCVD	< 1.8
BMI (kg/m^2^)	< 24.0

### Statistical methods

Statistical analysis was performed by means of SPSS 26.0 software, and the charts were drawn by SPSS 26.0 software or Graphpad 8.0. Kolmogorov–Smirnov test was used to check to normality of continuous variables. Group comparisons between CN group and CI group were performed using the one-way analysis of variance (ANOVA) (parametric variables) or Mann–Whitney U test (non-parametric variables) as appropriate. Mean ± standard deviation values applied to quantitative data that was normally distributed, while on the other hand, the median (quartile), i.e., M (P25-P75), applied to the quantitative data that was non-normally distributed. Categorical variables were compared using chi-square test. Multivariate logistic regression analyses were performed to identify major risk factors for incident cognitive dysfunction with T2DM. Significance was set for all comparisons at *P* < 0.05.

## Results

Over a period of 10 years, 504 eligible patients were in the final analysis. 44.44% of patients with T2DM suffered from cognitive impairment, while remaining 55.56% belonged to CN group. It just confused us that compared with CN group, there existed no statistically significant differences in HbA_1c_ (Z difference = -0.019, *P* = 0.985), duration of T2DM (Z difference = -0.268, *P* = 0.788), fasting blood glucose (Z difference = 0.465, *P* = 0.642), postprandial blood glucose (Z difference = 1.042, *P* = 0.297), admission glucose (Z difference = 0.513, *P* = 0.608), and remaining characteristics were shown in Table 2. These results remained consistent when target value of HbA_1c_ was set at 7.0, 7.5, 8.0 for the group comparison.

**Table 2:** Target items in cognitive impairment and T2DM management

Characteristics	CN group (*n* = 224)	CI group (*n* = 280)	F/χ^2^/Z	*P* value
HbA_1c_ (%)	7.2 (6.3, 8.2)	7.1 (6.4, 8.1)	-0.019	0.985
HbA_1c_ ≥ 7.0%, n (%)	110/191 (57.59%)	127/239 (53.14%)	0.851	0.356
HbA_1c_ ≥ 7.5%, n (%)	84/191 (43.98%)	95/239 (39.75%)	0.782	0.377
HbA_1c_ ≥ 8.0%, n (%)	56/191 (29.32%)	68/239 (28.45%)	0.039	0.844
Duration of T2DM (y)	6 (2, 11)	6 (1, 11)	-0.268	0.788
FBG (mmol/L)	8.4 (6.9, 10.7)	8.5 (7.0, 10.7)	0.465	0.642
FBG ≥ 7.0, n (%)	130/175 (74.29%)	189/243 (77.78%)	0.686	0.407
2-h PBG (mmol/L)	11.2 (9.6, 13.8)	11.7 (9.5, 14.4)	1.042	0.297
2-h PBG ≥ 10.0, n (%)	132/183 (72.13%)	167/238 (70.17%)	0.194	0.660
Admisson glucose (mmol/L)	9.5 (7.4, 13.2)	10.2 (7.2, 14.2)	0.513	0.608
SBP (mmHg)	138 ± 19	139 ± 20	0.195	0.649
DBP (mmHg)	80 (71, 88)	79 (71, 85)	-1.248	0.212
Total Cholesterol (mmol/L)	3.89 ± 0.91	3.86 ± 1.03	2.632	0.730
Triglyceride (mmol/L)	1.36 (1.03, 1.99)	1.39 (1.01, 1.91)	-0.022	0.983
HDL-C (mmol/L)	1.02 (0.86, 1.23)	1.00 (0.89, 1.19)	-0.384	0.701
LDL-C (mmol/L)	2.35 (1.71, 2.83)	2.22 (1.59, 2.79)	-0.754	0.451
Apo-A1 (g/L)	1.08 (0.96, 1.25)	1.06 (0.93, 1.21)	-1.181	0.238
Apo-B (g/L)	0.73 (0.59, 0.85)	0.71 (0.56, 0.88)	-0.879	0.380
BMI (kg/m^2^)	24.4 (23.1, 26.4)	24.5 (22.9, 26.6)	0.006	0.996
BMI stratification			3.336	0.503
Severely underweight, n (%)	0/190 (0)	1/235 (0.43%)		
Underweight, n (%)	2/190 (1.05%)	1/235 (0.43%)		
Normal weight, n (%)	45/190 (23.68%)	61/235 (25.95%)		
Overweight, n (%)	66/190 (34.74%)	67/235 (28.51%)		
Obesity, n (%)	77/190 (40.53%)	105/235 (44.68%)		

### Clinical outcomes

Patients in CI group with T2DM had a higher prevalence of brain infarction (|^2^ = 16.097, *P* = 0.000) and dementia (|^2^ = 23.124, *P* = 0.000) compared with patients in CN group with T2DM. Regarding HAMA score (Z difference = 2.017, *P* = 0.044), patients in CI group had higher scores at the baseline, but no significance was detected in incident anxiety disorder (|^2^ = 0.117, *P* = 0.732). In the matter of education level (Z difference = -2.668, *P* = 0.008) and severity of dependence (Z difference -5.085, *P* = 0.000), individuals in CN group tended to be better-educated and less dependent.

### Laboratory data

In point of items for liver function, globulin (Z difference = 2.116, *P* = 0.034), albumin (F difference = 0.291, *P* = 0.046), A/G (Z difference = -3.292, *P* = 0.001) were detected statistically significant. For the thyroid function, patients in CI group had a lower serum concentration in T3 (Z difference = -3.269, *P* = 0.001) and fT3 (Z difference = -4.160, *P* = 0.000). With regard to coagulation biomarkers, yet individuals in CI group had a higher serum concentration in D-dimer (Z difference = 3.211, *P* = 0.001) and fibrinogen degradation products (Z difference = 2.535; *P* = 0.011), while CI group reversely had a lower value of thrombin time (Z difference = -2.564; *P* = 0.010). Of note, patients in CI group had a higher serum concentration in HCY (Z difference = 2.030, *P* = 0.042), and lower creatine kinase (Z difference = -2.838; *P* = 0.005). Other parameters claimed to be associated with the cognitive dysfunction on the basis of articles published previously, were found no differences (Shown in Table 3).

**Table 3:** Demographic, clinical and laboratory characteristics of CN group VS CI group

Characteristics		CN group (*n* = 224)	CI group (*n* = 280)	F/χ^2^/Z	*P* value
Demographic & Clinical Features	Age(y)	63 (56, 70)	67 (61, 74)	4.735	**0.000**^**∗**^
	Gender, male, n (%)	156 (69.64%)	183 (65.36%)	1.038	0.308
	Residential area, village, n (%)	55 (24.55%)	83 (29.64%)	1.621	0.203
	Alcohol comsumption, n (%)	60 (26.79%)	59 (21.07%)	2.253	0.133
	Smoke, n (%)	75 (33.48%)	89 (31.79%)	0.163	0.686
	Hypertension, n (%)	152 (67.86%)	198 (70.71%)	0.479	0.489
	Coronary Heart Disease, n (%)	34 (15.18%)	53 (18.93%)	1.225	0.268
	Brain Infarction, n (%)	116 (51.79%)	194 (69.29%)	16.097	**0.000**^**∗**^
	Encephalatrophy, n (%)	12 (5.36%)	28 (10.00%)	3.671	0.055
	Dementia, n (%)	3 (1.34%)	36 (12.86%)	23.124	**0.000**^**∗**^
	Anxiety Disorder, n (%)	59 (26.34%)	70 (25.00%)	0.117	0.732
	Depression, n (%)	56 (25.00%)	63 (22.50%)	0.431	0.511
	Education level			-2.668	**0.008**^**∗**^
	Illiteracy, n (%)	7 (3.12%)	18 (6.43%)		
	Elementary school, n (%)	29 (12.95%)	54 (19.29%)		
	Junior high school above, n (%)	188 (83.93%)	208 (74.28%)		
Clinical Scale Tests	HAMA score	10 (7, 13)	11 (7, 15)	2.017	**0.044**^**∗**^
	HAMD score	10 (6, 13)	11 (7, 16)	1.215	0.224
	Severity of dependence (BI)			-5.085	**0.000**^**∗**^
	Self-care, n(%)	105 (46.88%)	79 (28.22%)		
	Mild dependence, n(%)	108 (48.21%)	157 (56.07%)		
	Moderate dependence, n(%)	10 (4.46%)	31 (11.07%)		
	Severe dependence, n(%)	1 (0.45%)	13 (4.64%)		
Inflammation Parameters	WBC (× 10^9/L)	5.87 (4.88, 7.01)	6.05 (4.94, 7.38)	1.058	0.290
	ANC (× 10^9/L)	3.61 (2.73, 4.24)	3.66 (2.69, 4.66)	0.881	0.378
	PLT (× 10^9/L)	183 (151, 221)	187 (152, 217)	0.096	0.923
	ESR (mm/hr)	14 (6, 26)	14 (8, 23)	0.359	0.719
Liver & Renal Function	ALT (IU/L)	19 (14, 27)	20 (13, 27)	-0.764	0.445
	AST (IU/L)	18 (14, 23)	18 (15, 23)	-0.164	0.870
	GGT (IU/L)	24 (17, 34)	24 (17, 36)	0.403	0.687
	TP (g/L)	66.0 ± 5.4	66.2 ± 5.7	0.001	0.695
	Glb (g/L)	25.5 (23.0, 28.4)	26.3 (23.5, 29.4)	2.116	**0.034**^**∗**^
	Alb (g/L)	40.1 ± 3.7	39.5 ± 3.5	0.291	**0.046**^**∗**^
	A/G	1.6 (1.4, 1.8)	1.5 (1.4, 1.7)	-3.292	**0.001**^**∗**^
	ALP (IU/L)	74 (60, 90)	77 (64, 93)	1.708	0.088
	Creatinine (umol/L)	84 (67, 100)	85 (68, 101)	0.351	0.726
	Uric Acid (umol/L)	272 (224, 331)	262 (215, 309)	-1.636	0.102
Thyroid Function	TSH (uIU/ml)	2.27 (1.54, 3.49)	2.11 (1.34, 3.15)	-1.241	0.215
	T4 (nmol/L)	95.24 (81.25, 111.08)	94.93 (80.95, 112.38)	-0.138	0.890
	fT4 (pmol/L)	16.20 (14.76, 17.91)	15.91 (14.06, 18.03)	-0.809	0.419
	T3 (nmol/L)	1.54 (1.36, 1.76)	1.44 (1.27, 1.61)	-3.269	**0.001**^**∗**^
	fT3 (pmol/L)	4.38 (3.98, 4.69)	4.13 (3.71, 4.53)	-4.160	**0.000**^**∗**^
	TPO-Ab (IU/ml)	16.24 (10.18, 36.78)	18.76 (10.11, 34.93)	0.154	0.878
	Thyroglobulin antibody (IU/ml)	15.00 (10.00, 26.10)	15.00 (10.00, 22.18)	-0.872	0.383
	Thyroglobulin (ng/ml)	7.32 (3.39, 13.73)	7.99 (3.42, 14.44)	0.115	0.909
Coagulation Markers	Prothrombin time (s)	10.70 (10.30, 11.20)	10.90 (10.40, 11.50)	1.528	0.127
	APTT (s)	24.95 (22.70, 27.98)	25.20 (23.10, 28.60)	0.961	0.337
	Fibrinogen (g/L)	2.76 (2.31, 3.26)	2.85 (2.40, 3.42)	1.534	0.125
	Thrombin time (s)	18.40 (17.40, 19.30)	17.90 (17.30, 19.00)	-2.564	**0.010**^**∗**^
	D-dimer (mg/L)	0.27 (0.19, 0.45)	0.33 (0.20, 0.62)	3.211	**0.001**^**∗**^
	FDP (ug/ml)	1.60 (1.20, 2.08)	1.73 (1.35, 2.42)	2.535	**0.011**^**∗**^
	Prothrombin activity (%)	97.30 (88.50, 105.80)	95.20 (86.70, 105.20)	-1.099	0.272
	International Normalized Ratio	0.95 (0.91, 0.99)	0.96 (0.91, 1.01)	1.285	0.199
Myocardial enzyme	LDH (IU/L)	171 (150,191)	173 (151, 194)	0.882	0.378
	LDH-1 (IU/L)	43 ± 14	43 ± 15	1.258	0.594
	CK (IU/L)	66 (51, 100)	60 (43, 81)	-2.838	**0.005**^**∗**^
	CK-MB (IU/L)	12 (10, 16)	12 (10, 14)	-0.287	0.774
	α-HBDH (IU/L)	139 (123, 157)	139 (124, 161)	0.667	0.505
	Homocysteine (umol/L)	10.87 (8.98, 14.12)	11.99 (9.28, 15.00)	2.030	**0.042**^**∗**^
	Vitamin B_12_ (pmol/L)	368.1 (235.4, 718.0)	363.6 (226.5, 629.6)	-0.776	0.438
	Folicacid (nmol/L)	15.10 (10.44, 22.19)	14.50 (9.33, 23.18)	-0.912	0.362
	NSE (ng/ml)	11.64 ± 2.76	11.14 ± 2.69	0.009	0.297

### Binary logistic regression analysis

Judging from the results of the univariate analysis above, binary logistic regression analysis was performed with age, education level, severity of dependence, the presence of brain infarction and dementia, globulin (Glb), albumin (Alb), A/G, triiodothyronine (T3), free triiodothyronine (fT3), D-dimer (D-Di), fibrinogen degradation products (FDP), thrombin time (TT), HCY, creatine kinase (CK) as independent variables and the incidence of cognitive impairment as the dependent variable. Eventually, we came to a conclusion that 6 parameters were significantly available to be dominant risk factors for the coexistence of cognitive dysfunction and T2DM (Shown in Table 4, all *P* < 0.05) as follows: HCY (OR = 2.048, 95%CI = 1.129–3.713), brain infarction (OR = 1.963, 95%CI = 1.197–3.218), dementia (OR = 9.430, 95%CI = 2.113–42.093), education level (OR = 0.605, 95%CI = 0.367–0.997), severity of dependence (OR = 1.996, 95%CI = 1.397–2.851), CK (OR = 0.514, 95%CI = 0.271–0.974). It turned out that protective factors ought to be education level and CK. Of note, individuals with T2DM and dementia took more prominently increased risk for cognitive dysfunction.

**Table 4:** Binary logistic regression analysis of risk factors for cognitive impairment and T2DM

Characteristics	Regression coefficient (β)	Standard error (SE)	Wald	*P* value	Odds ratio (OR)	95% confidence interval (CI)	sensitivity	specificity
HCY (umol/L)	0.717	0.304	5.571	0.018^*^	2.048	1.129–3.713	0.257	0.829
Brain infarction	0.675	0.252	7.152	0.007^*^	1.963	1.197–3.218	0.703	0.466
Dementia	2.244	0.763	8.643	0.003^*^	9.430	2.113–42.093	0.129	0.986
Education level	-0.503	0.255	3.894	0.048^*^	0.605	0.367–0.997	0.926	0.014
Dependence	0.691	0.182	14.436	0.000^*^	1.996	1.397–2.851	0.723	0.452
CK (IU/L)	-0.666	0.326	4.164	0.041^*^	0.514	0.271–0.974	0.035	0.959

It was worth mentioning that during the process of establishing logistic regression analytic models, the role of HCY was interfered by age. The interaction effect that age exerted would cover up the fact HCY took effect on cognitive function. After adjusting the interplay, it should be involved as age-related predictors for the incidence of T2DM-related cognitive dysfunction.

## Discussion

Subtle cognitive changes may take place at all stages of age with T2DM condition and progress slowly over time, whereas cognitive dysfunction and dementia lie in more severe stages of cognition, with progressive deficits, that predominantly disturb the aged [19]. In other words, people aged 45–65 also need to guard against cognitive decline, where middle-aged persons with T2DM had lower cognitive function [20]. The frail people of advanced age require a risk–benefit approach to management to attain content glycemic control and avert under-treatment or over-treatment. The adverse consequences that hypoglycaemia recurrently emerged could be massive when attempting to keep glycemic control rigorous [21]. Results from our study, inconsistent with the previous follow-up study supporting long-term dysglycemia being associated with faster cognitive decline during aging [22], intriguingly didn't support the hypothesis that profound cognitive deterioration could be obviously attributed to poor glycemic management. On the other hand, whether or not our study was able to characterize the occurrence of cognitive dysfunction rested with multiple factors, including the sample size, clinical comorbidity, and the sensitivity and specificity of the cognitive scale tested by.

**Fig. 2:**
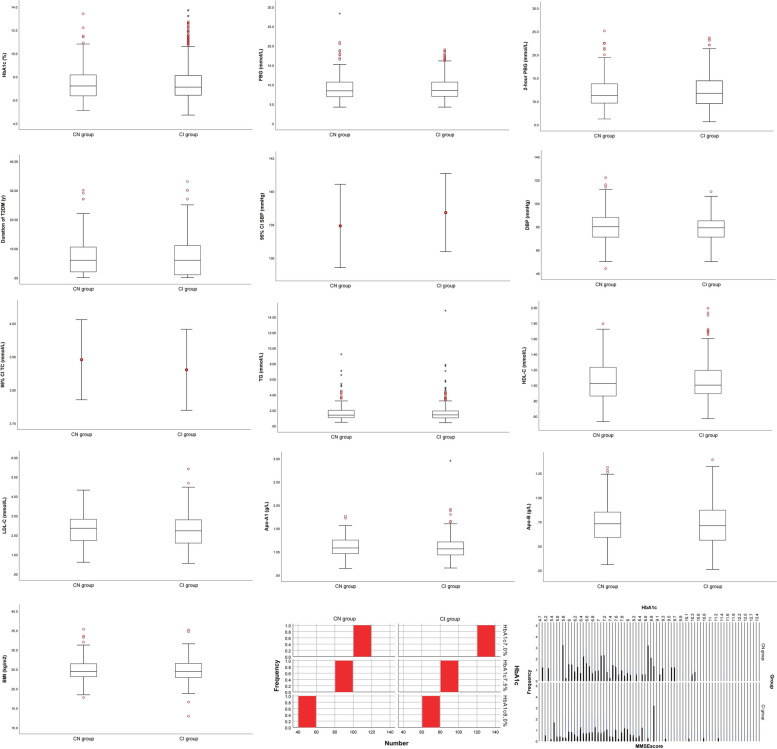
Descriptive graphs of target items for T2DM management. ^*^Abbreviations: Error Bar Chart applied to quantitative data that was normally distributed; Box Plot applied to the quantitative data that was non-normally distributed. All items in the figure made no significant differences. (all *P* > 0.05)

Taken together, Our results demonstrated that poor glycemic control might not absolutely trigger poorer performance on cognitive tests, but the presence of T2DM was still believed to be associated with cognitive dysfunction because the proportion of cognitive impairment was still dominant of the enrolled patients, and individuals with T2DM had a slightly higher rate of cognitive impairment, though not statistically significant. Nonetheless, our results illustrated that HbA_1c_-centered glycemic and lipid management seemed to play an unconspicuous role. As such, the value of HbA_1c_ was inclined to be lower in CI group, in all probability attributed to stringent glycemic control (Shown in Fig. 2). Therefore, it was speculated that glycemic control is still the best way to attempt to ameliorate frailty and physical impairment in the elderly, with or without DM [23].

As a rule, T2DM usually developed over a period of years, it was out of the question to precisely estimate the duration of glycemic excursions prior to a formal diagnosis. Although it was acknowledged there was an effect of duration of T2DM on magnitude of cognitive dysfunction, these group differences were conspicuously confounded by age [24]. Thus, HbA_1c_ might not be the best measure for capturing cognitive dysfunction in the elderly with T2DM. 1,5-Anhydroglucitol (1,5-AG), reflected additional information on glycemic instability and hyperglycemic excursions not reflected in HbA_1c_ or fasting blood glucose levels over a short period of time (2–14 days), which might be particularly relevant for cognitive dysfunction [25]. In addition to this, a previous study [26] illustrated that fructosamine and glycated albumin (GA), as two biomarkers that could better estimate short-term (2–3 weeks) glycemic control, were associated with incident dementia. However, a major shortcoming of our retrospective study was that no sufficient data on glycated albumin were available.

Results from our research, consistent with the essay recently published [27], revealed that deficiency in cognition was not significantly associated with body weight control and blood lipid regulation, ranging from obesity, overweight to obesity/overweight. Meanwhile, the serum level of dyslipidemia almost made no difference at baseline for the elderly with T2DM with vs without cognitive impairment. Even so, insulin resistance lied at a key junction that was influenced by obesity but also instigated multi-factorial downstream effects that at length manifest with metabolic and cognitive dysfunction [28]. In such cases, significant correlations were not observed between T2DM and cognitive performance, suggesting that the overlap might be specific to the illness and not seen with general obesity factors.

As for thyroid disorder, our study, consistent with the previous study [29], proved that thyroid function, the serum vitamin B_12_ and folic acid were not closely associated with cognitive dysfunction in older adults on the whole, and the interrelation was most likely a chance finding. However, since thyroid stimulating hormone (TSH) level distributions change noticeably with ageing, it was suggested that utilizing an age-specific TSH reference range and measuring fT4 or even fT3 levels might prevent potential hazard of cognitive consequences of over or under-treatment of thyroid disorders in the elderly population [30].

**Fig. 3:**
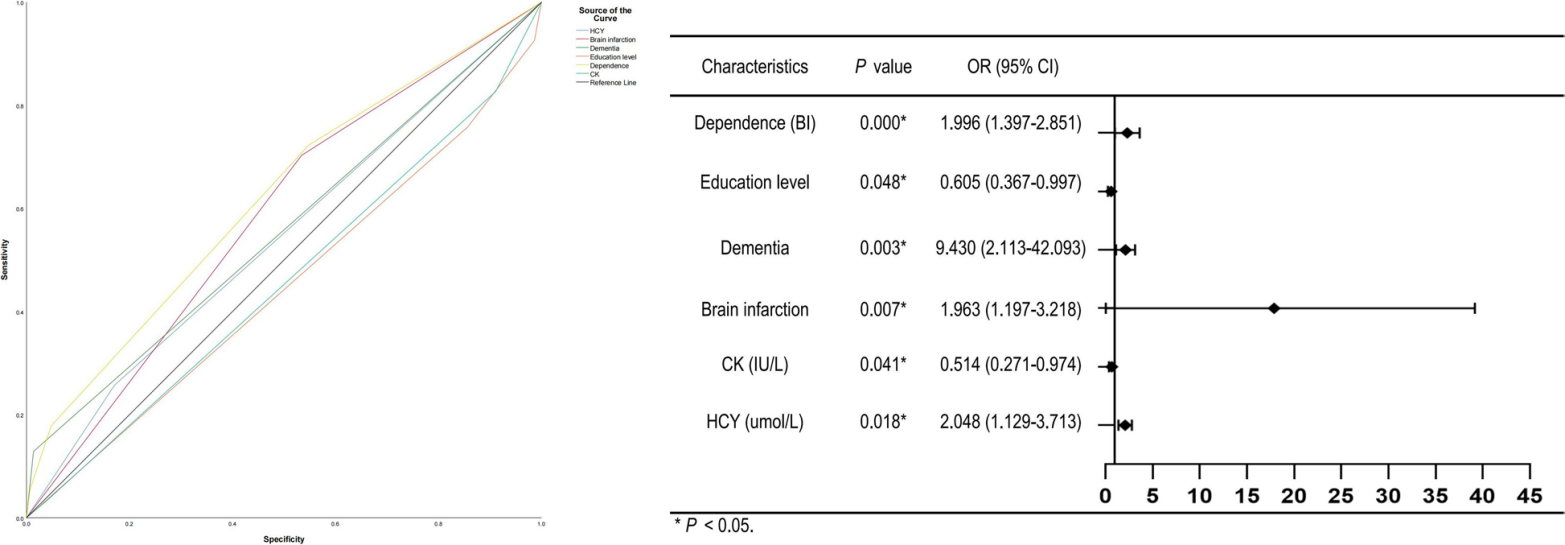
ROC curve and Forest Plot of risk factors for T2DM-related cognitive impairment

In a nutshell, receiver operator characteristic (ROC) curve and forest plot (Shown in Fig. 3) from our research demonstrated that patients of advanced age suffering from T2DM, to prevent cognitive dysfunction, clinicians should attach importance to the T2DM-related complications, principally aimed at incident brain infarction, dementia, higher serum concentration of age-related HCY, lower level of education, lower serum concentration of CK, and severity of dependence equally to physical inactivity. If these factors are determined to be causal, controlling them could minimize the degree of cognitive decline, and preventive countermeasures ought to think over ethno-regional differences [31]. With memory and executive function decline associated with dementia, older adults with comorbidity T2DM would be less compliant with the prescribed regimens, and hence had a tendency toward the weight loss and malnutrition [32], contributing to a higher risk of frailty. T2DM might accelerate cognitive decline indirectly via lower basal cortical thickness and reduction in brain reserve [33]. Accordingly, our results suggested that higher educational status, a proxy of cognitive reserve which might be beneficial to functional efficiency of the cognitive system [34], would protect against T2DM-related cognitive decline in the elderly. And brain infarction seemed to be sufficient to disentangle the robust relationship between T2DM and cognitive dysfunction, inconsistent with a large cross-sectional analysis of Canadians [35].

Surprisingly, our study detected the association between T2DM-related cognitive dysfunction and CK, which in all probability played a protective part with an up-regulation of serum level. Inconsistent with report previously, the down-regulation of CK used to be thought to be connected with neurodegenerative disorders, such as Alzheimer's disease [36]. CK was critically vital in cerebral energy requirements and it was susceptible to oxidative stress [37], which could be a potential mechanism of pathophysiology associated with cognitive dysfunction. Given the importance of CK as a potential biomarker for cognitive dysfunction, it might provide a novel approach to perfect detection of diagnostic sensibility.

A recent finding suggested that higher concentration of serum HCY and the presence of DM synergistically aggravated cerebral injury through increasing oxidative stress and neuroinflammation contributing to neuronal death and cerebrovascular burden [38], which was irrelevant to Alzheimer's disease and cerebrovascular disease. Strong evidence also had suggested that lowering HCY had major cognitive benefits and HCY lowering with b vitamins (mainly vitamin B_12_) altered the development of cognitive decline [39]. To sum up, hyperhomocysteinemia, susceptible to age [40], was bound to be a key risk factor for coexistence of T2DM and cognitive dysfunction of older adults.

More definitive answers will emerge from prospective cohort studies of large sample size that includes long follow-up periods. Novel fluid biomarker detection methods, such as the ultrasensitive single molecule array, open more and more opportunities to identify biomarkers to define and monitor the causes of cognitive decline and dementia with T2DM [10]. Perhaps in the future cerebrospinal fluid biomarkers with blood­based biomarkers will be widespread in a research setting and their value in clinical evaluation shall be explored. To avoid hypoglycaemia and speculate on potential anti-diabetic agents are still cornerstones in practice. In particular, sodium-glucose cotransporter 2 (SLGT2) inhibitors, the most viable oral anti-diabetic drugs to maintain optimal glycemic control, may be beneficial to cognitive improving in older adults with frailty associated with T2DM [41], probably by attenuated mitochondrial Ca^2+^ overload and reduced mitochondrial oxidative stress in endothelial cells [42].

The present study has a couple of strengths and limitation. To date, no studies have shown a correlation between creatine kinase and cognitive dysfunction. Thus, prospective study in the future may take into consideration that there exist potential creatine kinase and cognition, since creatine kinase was thought to be related to Huntington's disease. Nevertheless, there were still data gaps in this study, with the number of cases insufficient, and essential risk factors (such as glucose peak, fructosamine, glycated albumin, incidence of hypoglycemia, and diabetes medication) were not included in the medium limit of this study.

## Conclusion

In a nutshell, this study can provide a mind map for clinicians, and this study highlights a synergistic interaction between HCY, T2DM, and cognitive dysfunction. This is certainly a research topic that merits further investigation, particularly as more people develop T2DM at an earlier age, and hence experience longer exposure to chronically elevated glycemic values and vascular events. It is suggested cognitive reassessment should carry out annually to ensure an indispensable diagnosis is not passed up for patients of advanced age above 65-year-old. It is of particular concern that policy responses and interventions designed to preserve cognitive dysfunction in T2DM condition should be initiated preferably in middle-age adulthood and be sustained across a lifespan. Whereas, major risk factors, especially CK which played a protective role, should be paid more attention to T2DM-related cognitive dysfunction to assist physicians to prevent.

## Data Availability

The dataset generated and/or analyzed during the current study are not publicly available to protect the privacy of the respondents but are available from the corresponding author on reasonable request.

## Abbreviations

ASCVD: Arteriosclerotic cardiovascular disease
CN: Cognitively normal
CI: Cognitively impaired
FBG: Fasting blood glucose
PBG: Postprandial blood glucose
Apo-A1: Apolipoprotein A1
Apo-B: Apolipoprotein B
TPO-Ab: Thyroid peroxidase antibody
APTT: Activated partial thromboplastin time
FDP: Fibrinogen degradation product
NSE: Neuron specific enolase
